# Easily misdiagnosed X-linked adrenoleukodystrophy

**DOI:** 10.1186/s13052-024-01669-y

**Published:** 2024-07-02

**Authors:** Qiu-Hong Wang, Yang-Yang Wang, Jing Wang, Li-Ying Liu, Jing Gao, Guo-Zhen Hao, Chen Chen, Qian Lu, Shuo Dun, Qi Zhang, Li-Ping Zou

**Affiliations:** 1 Senior Department of Pediatrics, the Seventh Medical Center of Chinese PLA General Hospital, Beijing, 100010 China; 2 Medical School of Chinese PLA, Beijing, 100853, China; 3 Xuanwu Hospital Capital Medical University, Beijing, 100053 China; 4 Beijing Institute for Brain Disorders, Center for Brain Disorders Research, Capital Medical University, Beijing, 100069 China

**Keywords:** ABCD1, Addison, Adrenoleukodystrophy, Hit, Infection

## Abstract

**Background:**

Addison’s disease and X-linked adrenoleukodystrophy (X-ALD) (Addison’s-only) are two diseases that need to be identified. Addison’s disease is easy to diagnose clinically when only skin and mucosal pigmentation symptoms are present. However, X-ALD (Addison’s-only) caused by *ABCD1* gene variation is ignored, thus losing the opportunity for early treatment. This study described two patients with initial clinical diagnosis of Addison’s disease. However, they rapidly developed neurological symptoms triggered by infection. After further genetic testing, the two patients were diagnosed with X-ALD.

**Methods:**

We retrospectively analyzed X-ALD patients admitted to our hospital. Clinical features, laboratory test results, and imaging data were collected. Whole-exome sequencing was used in molecular genetics.

**Results:**

Two patients were included in this study. Both of them had significantly increased adrenocorticotropic hormone level and skin and mucosal pigmentation. They were initially clinically diagnosed with Addison’s disease and received hydrocortisone treatment. However, both patients developed progressive neurological symptoms following infectious disease. Further brain magnetic resonance imaging was completed, and the results suggested demyelinating lesions. Molecular genetics suggested variations in the *ABCD1* gene, which were c.109_110insGCCA (p.C39Pfs*156), c.1394–2 A > C (NM_000033), respectively. Therefore, the two patients were finally diagnosed with X-ALD, whose classification had progressed from X-ALD (Addison’s-only) to childhood cerebral adrenoleukodystrophy (CCALD). Moreover, the infection exacerbates the demyelinating lesions and accelerates the onset of neurological symptoms. Neither the two variation sites in this study had been previously reported, which extends the *ABCD1* variation spectrum.

**Conclusions:**

Patients with only symptoms of adrenal insufficiency cannot be simply clinically diagnosed with Addison’s disease. Being alert to the possibility of *ABCD1* variation is necessary, and complete genetic testing is needed as soon as possible to identify X-ALD (Addison’s-only) early to achieve regular monitoring of the disease and receive treatment early. In addition, infection, as a hit factor, may aggravate demyelinating lesions of CCALD. Thus, patients should be protected from external environmental factors to delay the progression of cerebral adrenoleukodystrophy.

**Supplementary Information:**

The online version contains supplementary material available at 10.1186/s13052-024-01669-y.

## Background

Addison’s disease is a primary adrenal insufficiency. Its typical causes are autoimmune diseases, infections, tumors, and genetic diseases. Congenital adrenal hyperplasia is the most common genetic defect. When symptoms of adrenal insufficiency, such as skin and mucosal pigmentation, are present, a patient can be easily clinically diagnosed with Addison’s disease, and corticosteroid supplementation is given [1, 2]. However, another important genetic disease behind the symptoms of adrenal insufficiency is X-linked adrenoleukodystrophy (X-ALD). X-ALD can only show the symptoms of adrenal insufficiency, that is, X-ALD (Addison’s-only) [3, 4].

X-ALD is caused by an ATP-binding cassette protein subfamily D1 (*ABCD1*) gene variation, which encodes peroxisomal ATP-binding cassette-transporter adrenoleukodystrophy protein (ALDP). The incidence of X-ALD is approximately 1/20,000. ALDP is responsible for transporting very-long-chain fatty acids (VLCFA) to peroxisome for β-oxidation. The dysfunctional *ABCD1* causes the failure of VLCFA degradation, thus leading to the accumulation of VLCFA in tissues and plasma and mainly involving cerebral white matter, the adrenal cortex, and the spinal cord [5, 6]. The excessive VLCFA leading to the production of reactive oxygen species (ROS) and redox imbalance. In turn, these two effects can lead to oxidative stress, inflammation, and finally manifested as progressive inflammatory demyelination of the brain, axonal degeneration, and adrenal insufficiency [7–10]. Phenotypic variability has a wide range in clinical manifestations, including childhood cerebral adrenoleukodystrophy (CCALD), adolescent cerebral ALD, adult cerebral ALD, adrenomyeloneuropathy, Addison’s-only, heterozygous, and asymptomatic [3, 7]. X-ALD (Addison’s-only) usually occurs after three years of age and mainly manifested as skin and mucosal pigmentation without neurological symptoms, and it may be the first or only symptom of ALD [1, 11]. As the disease progresses, some patients may develop cerebral ALD, which presents cerebral inflammatory demyelination and neurological symptoms [3, 4, 12, 13]. CCALD is the most common and serious phenotype with onset at about four to eight years of age and is manifested as rapid progressive cerebral inflammatory demyelination, which can cause death within a few years after the onset of symptoms. The treatment options are limited and the treatment window is narrow [11, 14–16]. For patients with only adrenal insufficiency, they tend to be clinically diagnosed with common Addison’s disease but not X-ALD (Addison’s-only). However, if X-ALD is not recognized and monitored in time, once it progresses to cerebral ALD and neurological symptoms occur, the time window for early treatment is easily lost due to the rapid progression of the disease.

Although X-ALD is a monogenic genetic disease, it has significant genetic heterogeneity. Even identical twins can manifest different clinical phenotypes, thus suggesting the role of environmental factors in the pathogenesis of X-ALD with different phenotypes [17–19]. Reports have demonstrated that the destruction of the blood-brain barrier (BBB) is crucial for the onset of cerebral ALD. *ABCD1* variation itself can increase the permeability of brain microvascular endothelial cells and alter white matter microvascular perfusion. More importantly, the brain microvascular endothelial cells are more sensitive to *ABCD1* variation under the condition of inflammation [20–22]. In addition, head trauma can reportedly initiate the onset of cerebral ALD, which shows rapidly progressive inflammatory demyelination at the site of brain contusion. That is, head trauma, as an external environmental factor, can be a triggering factor of disease progression [18, 19]. These circumstances highlight the role of environmental factors, such as inflammation and head trauma, in the onset and progression of X-ALD.

In this study, we report two patients who were initially clinically diagnosed with Addison’s disease. They developed rapid neurological symptoms induced by infection and were eventually diagnosed with CCALD after further genetic testing, strongly implicating the easy misdiagnosis of X-ALD and the role of infection in disease progression.

## Methods

### Patients

We retrospectively analyzed patients with X-ALD admitted to Chinese PLA General Hospital. We collected their clinical data, including clinical features, laboratory test results, imaging data, and molecular genetic results. In addition, we obtained informed consent from the patient’s legal guardian.

### Molecular genetics

Genomic DNA was extracted from the whole blood of the patients and their parents. Whole-exome sequencing was used for sequencing, and Sanger sequencing was used for validation. Variants were screened on the basis of genetic patterns, variation types, population frequencies, and a list of genes associated with the main phenotypic characteristics of the patients. We used the bioinformatics protein function prediction software to evaluate the pathogenicity of the variations. The classification of pathogenicity was performed using American College of Medical Genetics and Genomics (ACMG) guidelines.

## Results

The two patients enrolled in this study were initially clinically diagnosed with Addison’s disease due to skin and mucosal pigmentation. The infection accelerated the appearance of neurological symptoms, and further imaging examination and genetic detection confirmed them as CCALD.

### Clinical data

**Fig. 1:**
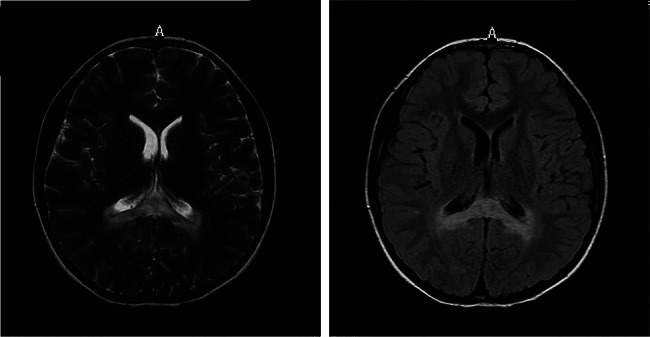
Brain MRI (patient 1): The long T2 signal intensity (left) and FLAIR image high signal intensity (right) on the splenium of the corpus callosum and the posterior side of the adjacent bilateral ventricles

**Fig. 2:**
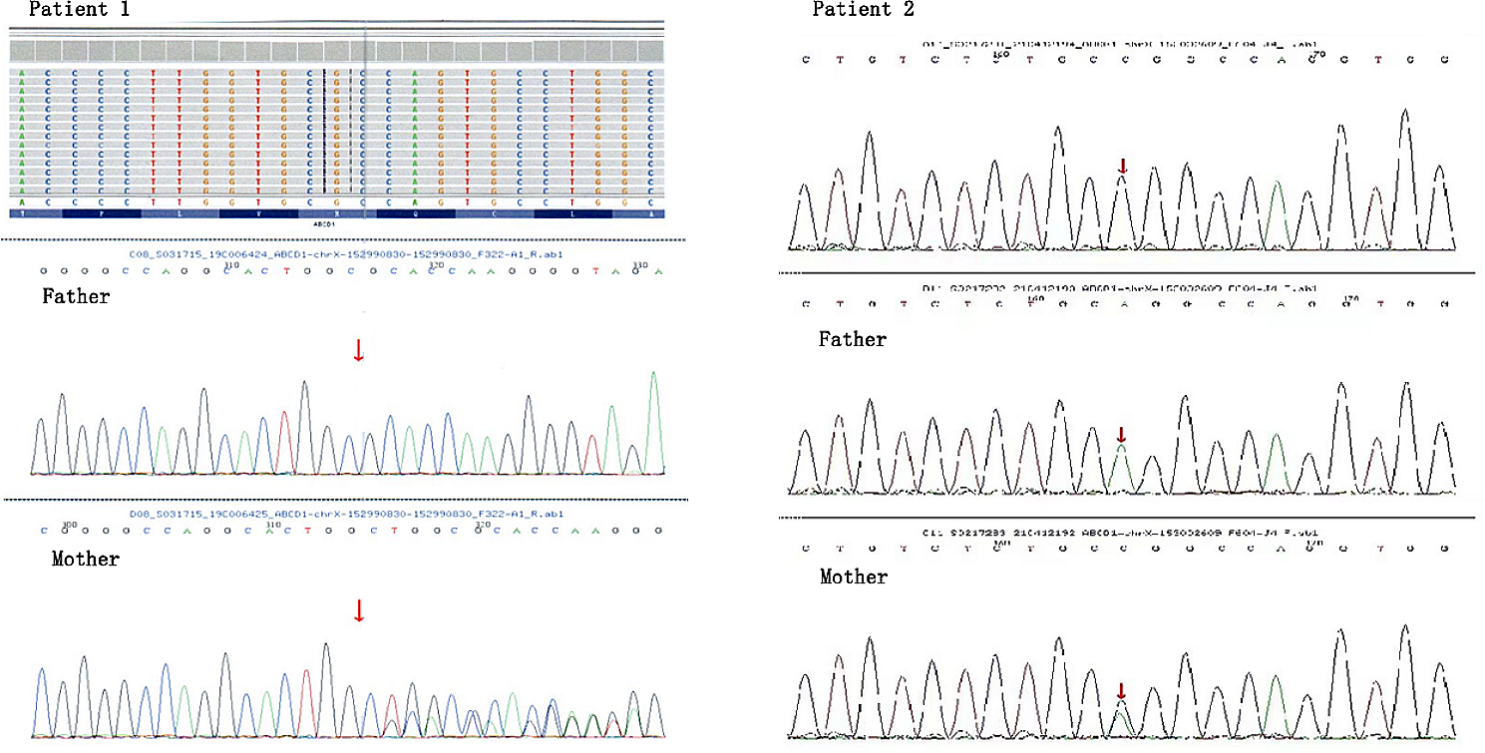
Variation sites of *ABCD1* gene in two patients. Patient 1, c.109_110insGCCA, p.C39Pfs*156(NM_000033); Patient 2, c.1394–2 A > C (NM_000033)

**Fig. 3:**
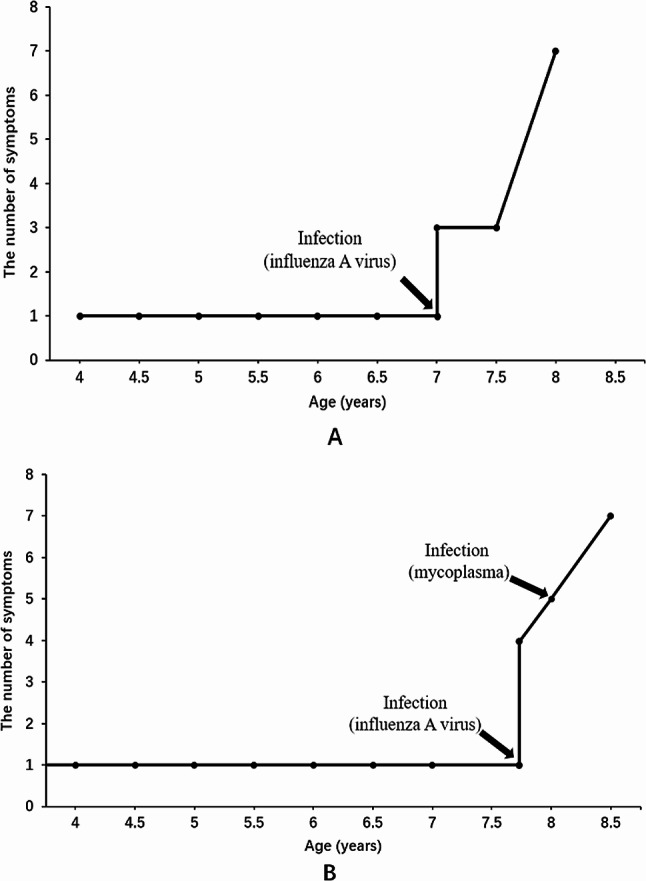
The changes in the clinical course of the two patients. (**A**): patient 1; (**B**): patient 2. The arrow indicates the point in time when the infection occurred. The ordinate uses the number of symptoms a patient has to represent the progression of the disease. At the age of four, patient 1 had only skin and mucosal pigmentation. At the age of seven, he rapidly developed neurological symptoms after infection. Patient 2 was born with skin and mucosal pigmentation and rapidly developed multiple neurological symptoms after infection at the age of seven years and nine months

Patient 1 was a male, 10-year-old individual, whose parents were not consanguineous. He began to develop systemic skin and mucosal pigmentation at the age of four. Adrenocorticotropic hormone (ACTH) at 8 am increased to > 1250 pg/ml (range 7.2–63.4 pg/ml), and cortisol at 8 am was 238.5 nmol/L (range 198.7-797.5 nmol/l). The patient was diagnosed with Addison’s disease and was treated with oral hydrocortisone (12.5 mg/d). After two months, ACTH decreased to normal level and remained normal for more than two years. The symptom of pigmentation was relieved, that is, the patient was in the stable stage during this period. However, at the age of seven, he acquired influenza A virus infection and experienced high fever (>39℃), which lasted for three days. Within days of infection, he developed unconventional thinking, attention-deficit disorder. ACTH at 8 am increased again, fluctuating between 114 and 766 pg/ml. On the basis of these neurologic symptoms, brain magnetic resonance imaging (MRI) was performed, and the results suggested long T1 and long T2 signal intensity, and high signal intensity on fluid-attenuated inversion recovery (FLAIR) image were observed near the splenium of the corpus callosum and the posterior side of the adjacent bilateral ventricles (Fig. 1). In addition, molecular genetics testing revealed *ABCD1* gene variation, c.109_110insGCCA (p.C39Pfs*156) (Fig. 2). Moreover, the patient was diagnosed with X-ALD (CCALD). That is, the patient was initially clinically diagnosed with Addison’s disease, and the onset of neurological symptoms was accelerated after the infection event. Then, he was eventually diagnosed with CCALD. As the disease progressed, at the age of eight, he developed irritability, mental retardation, poor balance, and impairment of vision. Furthermore, he had elevated levels of plasma VLCFA, C26: 2.4 nmol/ml (range ≤ 1.3 nmol/ml), C26/C22: 0.051 (range ≤ 0.023). The changes in the course of the disease are shown in Fig. 3.

Patient 2 was a male, eight-year-old individual, whose parents were not consanguineous. He had systemic skin and mucosal pigmentation since birth, especially the lips and joints. When he was four years old, he had a cardiac examination for evident pigmentation of the lips. No abnormality was found, and no further examination was conducted. ACTH at 8 am increased to > 2000pg/ ml (range 7.2–63.4 pg/ml), and cortisol at 8 am decreased to 14.3 nmol/l (range 198.7-797.5 nmol/l). He was initially diagnosed with Addison’s disease and given oral hydrocortisone (15 mg/d). Unfortunately, at the age of seven years and nine months, he developed a respiratory virus infection (etiology unknown) accompanied by fever (39.6℃). A few days after infection, he showed lagged response, poor comprehension, and exotropia. On the basis of these neurological symptoms, he completed a brain MRI scan. The results suggested multiple abnormal signals in the brainstem and the posterior horn of bilateral ventricles, considering demyelinating lesions. In addition, molecular genetics testing demonstrated *ABCD1* gene variation, c.1394–2 A > C (Fig. 2). Therefore, he was definitively diagnosed with X-ALD (CCALD). Overall, the patient was initially clinically diagnosed with Addison’s disease. However, after experiencing infection, disease progression accelerated, and neurological symptoms developed. Then, he was eventually diagnosed with CCALD. As the disease progressed, at the age of eight, he gradually developed distinct spastic gait. After another mycoplasma infection (38.6℃), within a few days, he suffered a rapid decline in visual acuity, inability to distinguish directions, and impaired verbal communication. Physical examination showed positive Romberg sign, positive Babinski sign, and poor performance in the finger-to-nose test. Moreover, he had elevated levels of plasma VLCFA, C26: 3.15 nmol/ml (range ≤ 1.3 nmol/ml), C24/C22: 1.62 (range ≤ 1.39), C26/C22: 0.064 (range ≤ 0.023). The changes in the course of the disease are shown in Fig. 3.

### Molecular genetic results

The results of the whole-exome sequencing of the two patients were *ABCD1* variation, and the variation sites were inherited from their mother. The variation site of patient 1 was c.109_110insGCCA(p.C39Pfs*156) (NM_000033), frameshift variation. The result of trap score was 0.464, and dbsSNV was suggested to be deleterious. ACMG was classified as pathogenic (PVS1 + PM2 + PP4). The variation site of patient 2 was c.1394–2 A > C (NM_000033), splice site variation. The prediction result of mutation taster was disease causing, and ACMG was classified as pathogenic (PVS1 + PM2 + PP4) (Table 1). These two variation sites were not reported in ClinVar and HGMD databases, which expanded the genetic spectrum of *ABCD1* gene variation.

**Table 1:** Genetic variation sites of the two patients (NM 000033)

Patient	Gene	Origin	Location	Variation site	Amino acid	Variation type	Variation taster	Trap Score	dbsSNV	ACMG classification
1	ABCD1	maternal	Exon 1	c.109_110insGCCA	p.C39Pfs*156	frameshift variation	disease causing	—	—	pathogenic
2	ABCD1	maternal	IVS 4	c.1394–2 A> C	—	splice site variation	—	0.464	Deleterious	pathogenic

## Discussion

This study reported two patients with X-ALD, whose initial symptoms were adrenal insufficiency and were clinically diagnosed with Addison’s disease (primary adrenal insufficiency). However, the possibility of X-ALD (Addison’s-only) was ignored, and the disease was not monitored in time. Neurological symptoms after infection led to further brain MRI and genetic testing, and the two patients were finally diagnosed with X-ALD (CCALD). As demyelinating lesions may not appear rapidly within days of infection, the patients may have progressed to CCALD prior to infection, and the infection may have exacerbated the inflammatory demyelination of CCALD and triggered the emergence of neurological symptoms. This study highlights the importance of genetic testing in patients with adrenal insufficiency and the role of infection in promoting cerebral ALD.

X-ALD is peroxisome disease caused by *ABCD1* variation. Its clinical phenotypic severity varies greatly, with mild cases presenting only adrenal insufficiency, that is, Addison’s-only, and severe cases presenting rapidly progressive CCALD [3, 7]. Adrenal insufficiency can be the first clinical manifestation of X-ALD. For CCALD, it may only present as adrenal insufficiency before neurological symptoms [4, 23–25]. During this period, given that only adrenal insufficiency symptoms were present, such as skin and mucosal pigmentation, but no neurological symptoms, it was easy to be simply diagnosed as Addison’s disease (primary adrenal cortical insufficiency). Meanwhile, the possibility of *ABCD1* variation-related X-ALD (Addison’s-only) was ignored, especially the risk of progression to cerebral ALD [1, 26–28]. In this study, two patients initially had only skin and mucosal pigmentation. They were clinically diagnosed with Addison’s disease and only received symptomatic treatment with hydrocortisone. Given its incomplete genetic testing, the existence of X-ALD (Addison’s-only) was ignored. Therefore, skin and mucosal pigmentation is also an early warning symptom of X-ALD. Thus, the possibility of X-ALD must be considered, and genetic testing must be completed as soon as possible.

For CCALD, before the onset of clinical symptoms, the earliest brain abnormalities are demyelinating lesions confined to brain MRI, which means the patient is in a presymptomatic stable state. Neurologic symptoms may occur when inflammatory demyelination progress. Once neurological symptoms appear, the disease progresses rapidly, and the presymptomatic stable state is difficult to maintain. Affected patients may have difficulty in communication, spasmodic gait. Eventually, patients may develop major functional dysfunction (MFD), such as being bedridden, blind, unable to speak or respond, fed by gastrostomy; moreover, death usually occurs two to four years after onset of symptoms [29]. Therefore, delaying the onset of neurological symptoms is critical to stabilizing the condition. In this study, after the infection of influenza A virus, the ACTH level of patient 1, which had dropped to normal, increased again and gradually manifested neurological symptoms of CCALD. Patient 2 also presented with neurological symptoms after respiratory virus infection. Consistently, both patients presented an onset of neurological symptoms within days of infection. This fulminant clinical process is mainly associated with the aggravation of inflammatory demyelinating lesions in the white matter [18, 19]. The two patients underwent brain MRI after neurological symptoms, and the scan indicated demyelinating lesions. As demyelinating lesions of the brain may not appear rapidly within a few days after infection, the demyelinating lesions on brain MRI may have existed before infection. Both patients had progressed to CCALD prior to infection and were in a pre-symptomatic stable state (only demyelinating lesions in head MRI). The infection event aggravated the demyelinating lesions and accelerated the appearance of neurological symptoms. Considering the role of environmental factors in the pathogenesis of X-ALD [18, 19], the experiences of these two patients suggest that infection, as a triggering factor, may increase the risk of triggering progression of inflammatory demyelinating in patients with CCALD.

We further analyzed the possible pathological basis of CCALD triggered by infectious factors. VLCFA-associated oxidative stress in the brain leads to inflammatory demyelination in patients with *ABCD1* variations [8, 9]. Similarly, after infection, given increased body metabolism, hyperactive mitochondrial metabolism produces more ROS, which may increase white matter susceptibility to oxidative stress [8]. In addition, infections, such as viral infections, can activate the immune response and promote the release of inflammatory factors [30]. So, infection-induced oxidative stress and inflammation may be important factors in accelerating the progression of inflammatory demyelination. In addition, considering the higher vulnerability of brain microvascular system to *ABCD1* deficiency in the setting of inflammation [20–22], the inflammatory response caused by viral infection may further increase BBB permeability and microvascular flow heterogeneity, thus leading to the infiltration of peripheral inflammatory cells and brain parenchymal damage. Therefore, for patients with CCALD, infection, as a hit factor, promotes the progression of inflammatory demyelination of the brain. For CCALD, after early diagnosis, patients should be protected as extensively as possible to avoid infection, head trauma, and other events, which can stabilize the disease to a certain extent, to reduce or delay its progression to MFD or death.

Hematopoietic stem cell transplantation (HSCT) or hematopoietic stem cell gene therapy can effectively delay the progression of neurological diseases only in the early stage of CCALD [16, 31]. More importantly, better clinical outcomes can be achieved by receiving HSCT before the onset of neurological symptoms [11]. On this basis, neonatal ALD screening has become popular in recent years [32, 33]. Aubourg et al. recommend that any boy or adult male with Addison’s disease must be tested for ALD, given the genetic counseling, and the potential benefits of therapeutic intervention [34]. Given the prognostic implications of undiagnosed ALD, genetic testing should be performed as soon as possible for patients with early adrenal insufficiency related symptoms to achieve early diagnosis of ALD and improve the general outcome of these patients. If a definite diagnosis of X-ALD is made, brain MRI should be monitored regularly. Once inflammatory demyelinating lesions associated with cerebral ALD occur, treatment should be given as soon as possible. In addition to early treatment, this study also emphasizes early prevention before the onset of neurological symptoms, that is, avoiding the occurrence of hit events, such as infection, to delay the progression of the disease.

## Conclusions

This study highlights the importance of early genetic detection by reporting two patients initially diagnosed with Addison’s disease (primary adrenal insufficiency) and ignoring the presence of X-ALD (Addison’s-only). In addition, this study highlights the role of infection in accelerating the progression of inflammatory demyelination in the brain, which causes the onset of neurological symptoms. For patients with early definite diagnosis of CCALD, infection and other external events should be avoided as much as possible to maintain the stable state of the disease to reduce or delay its progression to MFD or death. Two new variation sites, c.109_110insGCCA(p.C39Pfs*156), c.1394–2 A > C, have extended the genetic variation spectrum.

### Electronic supplementary material

Below is the link to the electronic supplementary material.

Supplementary Material 1

## Data Availability

All data generated or analyzed during this study are included in this published article.

## Abbreviations

X-ALD: X-linked adrenoleukodystrophy
CCALD: Childhood cerebral adrenoleukodystrophy
ALDP: Adrenoleukodystrophy protein
VLCFA: Very-long-chain fatty acids
ROS: Reactive oxygen species
BBB: Blood-brain barrier
HSCT: Hematopoietic stem cell transplantation
ACMG: American College of Medical Genetics and Genomics
ACTH: Adrenocorticotropic hormone
MRI: Magnetic resonance imaging
