# Supplementary material for: Easily misdiagnosed X-linked adrenoleukodystrophy
Source: Ital J Pediatr. 2024 Jul 2;50:124. doi: 10.1186/s13052-024-01669-y (PMC11218101; doi:10.1186/s13052-024-01669-y)
Supplement: Supplementary file 1 — Supplementary Material 1 [file 13052_2024_1669_MOESM1_ESM.docx]

Dear editors:

We are sending a manuscript entitled “Easily misdiagnosed X-linked adrenoleukodystrophy”, which we would like to submit for possible publication in Italian Journal of Pediatrics.

We believe that the paper may be of particular interest to the readers of your journal because we suggest that for patients with only symptoms of adrenal insufficiency, it cannot be simply clinically diagnosed as Addison's disease. It is necessary to be alert to the possibility of ABCD1 variation, and complete genetic testing as soon as possible to identify X-ALD (Addison's-only) early, so as to achieve regular monitoring of the disease and receive treatment early. In addition, infection, as a hit factor, may accelerate the progression of inflammatory demyelination in the brain, as a result, causing the onset of neurological symptoms. For patients with early definite diagnosis of X-ALD, infection and other external events should be avoided as far as possible to maintain the stable state of the disease, so as to reduce or delay its progression to MFD or death.

Although the influence of environmental factors on ALD has been mentioned in the literature, there is no clear report on infection as an environmental trigger, especially infection is a common disease in children. We think that it is the neglect of common diseases that makes the specific children with ABCD1 gene mutation aggravated under the trigger of multiple infections, and finally showed neurological symptoms.

In addition, hematopoietic stem cell transplantation and hematopoietic stem cell gene therapy can only delay the disease progression of CCALD patients in the early stage of the disease, so considering the short treatment time window of CCALD, we emphasized that early management and prevention are very important to control the progression of the disease.

All authors have read and approve this version of the article, and due care has been taken to ensure the integrity of the work. This paper has not been published or submitted elsewhere. No conflict of interest exits in the submission of this manuscript. We would appreciate your consideration of this manuscript for publication as an original article in Italian Journal of Pediatrics, and we look forward to receiving comments from the reviewers.

Sincerely yours，

Li-Ping Zou Professor

Faculty of Pediatrics, Chinese PLA General Hospital;

Department of Pediatrics, The First Medical Center of Chinese PLA General Hospital

100853 Beijing, China,

Tel: +86-10-55499016;

Fax: +86-10-66939770;

E-mail: [zouliping21@hotmail.com](mailto:zouliping21@hotmail.com)
